# Evaluating the sustainability of a three-year community-based intergenerational project in Hong Kong: a longitudinal mixed methods study from the perspective of service providers

**DOI:** 10.1186/s43058-026-00920-3

**Published:** 2026-04-15

**Authors:** Eliza Lai-Yi Wong, Dorothy Yingxuan Wang, Nelson Chun-Yiu Yeung, Phoenix Kit-Han Mo, Per Nilsen, Carol Ka-Po Wong, Samuel Yeung-Shan Wong, Eng-Kiong Yeoh

**Affiliations:** 1 JC School of Public Health and Primary Care, Faculty of Medicine, The Chinese University of Hong Kong, Hong Kong SAR, China; 2 Centre for Health Systems and Policy Research, JC School of Public Health and Primary Care, Faculty of Medicine, The Chinese University of Hong Kong, Hong Kong SAR, China; 3 Division of Community Medicine, Department of Medical and Health Sciences, Linköping University, Linköping, 581 83 Sweden

**Keywords:** Intergeneration, Sustainability capacity, Community-based, Barriers and facilitators, Implementation sciences

## Abstract

**Background and objectives:**

Intergenerational programmes improve older adults’ cognitive, emotional, social, and health outcomes. However, their long-term sustainability remains unclear, which is important for ongoing effectiveness. This study aimed to evaluate the sustainability of a three-year community-based intergenerational project and the underlying facilitators and barriers from service providers’ perspectives.

**Research design and methods.:**

A longitudinal, mixed-methods approach was applied. The Program Sustainability Assessment Tool (PSAT), consisting of eight domains, was used to assess the project’s sustainability capacity, which is the ability of a programme to maintain its operations, benefits and impacts over time. Barriers and facilitators in PSAT domains were determined by the mean value of each domain. Semi-structured interviews were further administered to identify implementation determinants. A total of 28 managerial and front-line staff responsible for the programme were recruited from three local non-profit organisations. They completed baseline and three rounds PSAT survey and three focus-group interviews across the three-year project. Descriptive statistics and content analysis were conducted for quantitative and qualitative data, respectively.

**Results:**

The total sustainability scores during the three-year implementation of the programme were 5.16 ± 0.65 in baseline, 5.66 ± 0.54 in Year 1, 5.62 ± 0.67 in Year 2, and 5.18 ± 0.48 in Year 3. Domains as consistent facilitators across the three-year implementation were organisational capacity and programme evaluation, while domains as consistent barriers were partnerships and strategic planning. Environmental support, funding stability, programme adaptation, and communications were found as mixed impacts during three years of implementation.

**Discussion and implications:**

Service providers perceived the overall sustainability of the community-based intergenerational programme as acceptable. Fluctuations in sustainability reflected the life cycle of the programme. Factors affecting the long-term implementation are common practical issues for community-based programme and need to be addressed, especially in the aspects of funding stability, partnerships, and strategic planning, as well as those specific to intergenerational relationship and communication.

**Supplementary Information:**

The online version contains supplementary material available at 10.1186/s43058-026-00920-3.

Contributions to the literature

- This research is one of the few studies that assess the sustainability capacity of a community-based intergenerational project.
- The organizational capacity and program evaluation were found as consistent facilitators while the partnerships and strategic planning were identified as consistent barriers through the course of program life cycle.
- Critical determinants specific to intergenerational programs are knowledge and attitudes to the intergenerational relationship, synergy between younger and older participants, and program design.
- Capacity building workshop as a single implementation strategy may not enough to maintain sustainability capacity.

## Background

Intergenerational relationships and harmony are crucial to the well-being of young and older adults, primarily through the exchange of resources. Reciprocal resource transfers, including financial, instrumental and emotional support, can be realised through both familial and nonfamilial intergenerational interactions [1, 2]. These interactions foster relationship-building across generations, enhance cross-age attitudes, promote social connectedness, and encourage service activities, all of which contribute to older adults’ sense of purpose and a sense of value in life [2]. However, an increasing number of older adults live longer but often do so with only a spouse or alone. This trend can be attributed to declining birth rates, shifts in family values and economic pressures. Another observation is that the next generation tends to move out and may not live close together for various reasons. Thus, older adults tend to have weaker intergenerational relationships and support, resulting in poor health outcomes and higher hospitalization [3]. Family caregivers are one of the key pillars of the healthcare system to strengthen patient’s self-care and maintain their health.

In recent decades, intergenerational engagement has been increasingly integrated into public health interventions for older adults through various initiatives, including centre-based visit programmes, shared campus care, and family daycare [4]. These intergenerational programmes have demonstrated significant benefits for older adults’ cognitive, emotional, social, and health outcomes, including cognitive functioning, anxiety, depression, generativity, participation in physical and social activity, fitness improvements, and quality of life [5–8]. Moreover, studies have revealed unintended positive effects on younger participants, including a deeper understanding of older adults, a greater sense of self-worth, and an increased passion for volunteering [9–11]. This intergenerational engagement is important for fostering societal harmony and encouraging the younger generation to actively contribute to the healthcare services for older adults.The Hong Kong population has one of the longest life expectancies in the world, with over 1.45 million individuals aged 65 and older, representing 20.5% of the total population in 2021 [12]. This figure is expected to rise to 2.58 million, or 35% of the total population, by 2069 [13]. Alongside this demographic shift towards an ageing population, Hong Kong is also experiencing changes in family structure and traditional values. Unlike the historically tight-knit family relationships and strong support systems rooted in Confucian wisdom (family obligations, filial piety and harmony) [14, 15], modern family values are more affected by smaller household units, women’s participation in the labour market, and larger geographical distance [16, 17].

The modern values challenge the traditional capacity, sense of responsibility, and support across different familial generations and lead to a looser familial relationship [18, 19]. This also calls for more intergenerational aggregation, solidarity and cohesiveness across different generations, not only limited to family levels but also at a community level. To exert older persons’ expertise to serve society, impart wisdom and experience, and restore and strengthen positive intergenerational relationships and perceptions, Hong Kong government and different institutions have taken various initiatives, including lifelong learning programmes, Neighbourhood Active Ageing Project, and different community-based projects. One of them was a community-based project named “Jockey Club Generation Connect Project” (JCGCP), which was held from 2018–2021 among older and younger generations. These projects included different components to promote intergenerational understanding, interaction and support, and have shown effects on intergenerational harmony, solidarity and well-being [20, 21].

Despite existing evidence-based approaches highlighting the beneficial impact of support from younger generations on older adults’ well-being, implementation remains a challenge, especially regarding the long-term sustainability of intervention when relying on self-financing models after initial funding is secured in community settings. Therefore, evaluating the barriers and facilitators encountered during implementation is essential [22]. Sustainability capacity refers to the capacities, structures and processes that enable an organization to maintain its system and leverage resources for the effective implementation and continuance of evidence-based policies and activities over time [23, 24]. Planning and assessing the sustainability capacity of a programme has become increasingly important, recognized as a key element because it reflects the efficiency and effectiveness of resource allocation, which can affect future funding decisions for various health programmes [25, 26]. According to the Program Sustainability Assessment Tool (PSAT) evaluation framework, the sustainability capacity of an intergenerational programme should encompass aspects of environmental support, funding stability, partnerships, organisational capacity, programme evaluation, programme adaptation, communications, and strategic planning [23, 27, 28]. Assessing these factors can reliably capture the sustainability capacity of an intergenerational programme, and improving them can facilitate its continuity, spread, routinisation, and long-term implementation of the programme beyond its initial phase [29].

Given limited evidence on the sustainability of intergenerational programmes, this study aims to evaluate the sustainability capacity of a community-based intergenerational project in Hong Kong over three years. It will also identify the barriers and facilitators associated with this sustainability from the perspectives of service providers. Sustainability is as crucial as the adoption and implementation of such initiatives. The findings of this project can provide valuable insights to enhance the sustainability capacity of community programmes.

## Research design and methods

### Overview of the “Jockey Club Generation Connect Project” (JCGCP)

JCGCP was a three-year community-based project held from 2018 to 2021, funded by a donation, in collaboration with one university and three local non-governmental organisations (NGOs). JCGCP aimed to promote the intergenerational harmony, solidarity and well-being among older and younger generations in Hong Kong. A series of programmes based on social theories and empirical evidence were developed at the family, school-based and community levels, accordingly, including “Life Review” (LR), “Medical Visit Buddy” (MVB) and “Traditional Culture and Health Programmes” (TC). LR was a family-oriented programme using life review and wish fulfilment to connect older and younger family members. It provided opportunities for younger family members to review the lives of older members and to understand the older generation’s contribution to the family and society. MVB recruited undergraduates from the faculty of Medicine and Social Science. Students led health promotion activities with their partnering older adults through home visits and escort clinics. TC organised a variety of centre-based traditional activities (e.g., crafts, exercise, and sharing of old recipes) for different generations to experience intergenerational co-existence and collaboration. Each of the three programmes was held independently, and each round lasted for one year. In total, three rounds were held for each programme. The eligibility criteria for both older and younger participants in JCGCP were Chinese ethnicity, with normal cognitive function and communication ability. Older participants should be aged 60 or over, and those in the MVB programme should be diagnosed with at least one type of chronic disease. Finally, 614 older adult participants (LR: 114; MVB: 162; TC: 446) were recruited and participated in the programmes.

### Study design and participants

This study employed purposive sampling and a longitudinal, sequential mixed-methods approach to investigate service providers’ perspectives on the sustainability capacity of JCGCP. We used the Journal Article Reporting Standards for Mixed Methods Research to report our study (Appendix 1). Participants, mainly the programme organisers, managers and front-line staff from the three partner NGOs, were recruited in the capacity building workshops. These workshops were held annually during the project period and aimed to build and promote staff’s awareness and capacity to implement intergenerational programmes. A total of four rounds of workshops were conducted, focusing on the following components over 4 h: the design, operation, evaluation, and sustainability of the project, identifying related barriers and facilitators, and providing specific training and strategies. The attendance rate for four rounds of workshops was 100%. At the end of each annual workshop, attendees were invited to complete a survey on the project sustainability using the PSAT as the measurement tool [28]. To have an in-depth understanding of the facilitators and barriers of programme implementation and sustainability, these attendees were further invited for semi-structured individual interviews. Three rounds of in-depth interviews were eventually held after each of the three rounds of intervention between 2019 to 2021 as 2018 was the preparation phase of intervention.

### Instrument - Program sustainability assessment tool

Programme sustainability capacity was assessed by PSAT, a tool developed by the Center for Public Health Systems Science, Brown School, Washington University in Saint Louis and validated in 252 U.S. public health programmes (Cronbach’s α: 0.88) [27]. Sustainability capacity is the ability of a programme to maintain its operations, benefits and impacts over time. This capacity is influenced by 8 domains assessed with 40 items: (a) environmental support, (b) funding stability, (c) partnerships, (d) organizational capacity, (e) programme evaluation, (f) programme adaptation, (g) communications, and (h) strategic planning (Appendix 2).

Each of 40 items is rated based on a Likert scale from 1 (little or no extent) to 7 (a very great extent) points, or “Not able to answer”. Web-based questionnaire was used for assessment in our study, supplemented by paper-version available at www.sustaintool.org. Language adaption was not required as all respondents mastered English. The score of each domain was calculated by the mean score of the five individual items in that domain, and the overall score was finally calculated by the mean score of the eight domains. Domains and items higher than the overall rating were identified as facilitators, otherwise barriers. There were four rounds of assessments for the JCGCP, not only reflecting yearly performance of each domain, but also monitoring the changes of sustainability throughout the overall course of the project.

### Data collection and management

Four rounds of sustainability capacity assessment using PSAT were conducted. The baseline assessment was conducted in April 2018, before the commencement of the programme and the following three assessments were done after each round of the programme in May 2019, June 2020, and July 2021, respectively. Invitations attached with paperersion and web link of the questionnaire were sent to all attendees of the workshops. Completed questionnaires were collected and checked by a research assistant, then configured into a database in Microsoft Excel. Besides surveys, semi-structured individual interviews were conducted near the end of each programme round in January 2019, January 2020, and April 2021, respectively, as reviews of the current-year interventions. Interviews were conducted by trained research assistants face-to-face or via video conference. The interview audio was recorded and then transcribed verbatim. Written consent was obtained from all participants in the quantitative survey and semi-structured interview.

### Quantitative data analysis

For quantitative data, the mean score and standard deviation (SD) of each item, each domain and overall performance based on PSAT were presented. One-Way ANOVA (Analysis of variation) and pairwise multiple comparisons with Bonferroni correction were used to compare the PSAT scores across different years. Data analysis was performed using SPSS version 27 (SPSS, Inc., IBM, Armonk, NY).

### Qualitative data analysis

For qualitative data, perceived facilitators and barriers related to programme sustainability were identified by direct content analysis [30]. Facilitators were conditions that promoted or strengthened sustainability, and barriers were obstacles or challenges that hindered sustainability. Two researchers coded the transcripts independently, discussed the generated themes regarding barriers and facilitators, and ensured the convergence and divergence of the coding scheme. Different themes corresponding to barriers and facilitators were mapped to eight domains of PSAT. The final themes were reviewed by all other team members, and any disagreements were discussed to reach a consensus. NVivo was used to manage the qualitative data.

## Results

### Demographics

In total, 28 staff from three NGOs participated in the study, completing 54 quantitative survey responses and all of them subsequently participated in qualitative interviews. The response rate in the four rounds of the quantitative assessments was 61%, 100%, 88%, and 56%, respectively. The characteristics of the respondents are shown in Table 1. They took different roles in the project, including programme design, participant recruitment, activity organization, service delivery, administration and supervision.

**Table 1:** Characteristics of respondents (*n* = 28)

Characteristics	N	%
Partner institution		
NGO 1	8	28.6
NGO 2	11	39.3
NGO 3	9	32.1
Role in project^a^		
Frontline	15	53.6
Managerial	13	46.4
Program involved		
All activities	10	35.7
Tradition culture	11	39.3
Medical visit buddy	3	10.7
Life review	4	14.3
Number of rounds participated^b^		
1	12	42.9
2	9	32.1
3	4	14.3
4	3	10.7

^a^Frontline staff included a team member, project assistant, service officer, service assistant, and social worker; while managerial staff included centre leader, centre manager, chief officer, team leader, program manager, project coordinator, and project supervisor

^b^Respondents can attend one or more rounds of surveys and interviews

Fifteen respondents (53.6%) were frontline staff in the project (e.g., team member, project or service assistant, and social worker), and the remaining 13 (46.4%) were managerial staff (e.g., project leader, director, manager, officer, and coordinator). Of the four rounds of assessments, 12 staff (42.9%) attended only one, 9 staff (32.1%) attended two, 4 staff (14.3%) attended three and 3 of them (10.7%) attended all four rounds.

### Overall sustainability capacity

The sustainability capacity scores of JCGCP by items, domains, and overall performance across four years are presented in Table 2. Generally, the sustainability capacity of JCGCP by domain and by year was leaning towards positive sides (mean score > 4 of 7). In the baseline year, the overall score of sustainability capacity was 5.16 ± 0.65, and it significantly increased to 5.66 ± 0.54 in the programme year 1 (compared to the baseline year: P = 0.050), remained stable at 5.60 ± 0.67 in the year 2, and finally significantly dropped to 5.21 ± 0.50 in the final year compared to the second year (P = 0.025).

**Table 2:** PSAT score of JCGCP (2018–2021)

	**Round 1** **(2018)** **(*N* = 11)**	**Round 2** **(2019)** **(*N* = 18)**	**Round 3** **(2020)** **(*N* = 15)**	**Round 4** **(2021)** **(*N* = 10)**
**Overall score**	**5.16 ± 0.65**	**5.66 ± 0.54**^a^	**5.62 ± 0.67**	**5.21 ± 0.50**^b^
**a) Environmental Support**	**5.11 ± 0.74**	**5.65 ± 0.92**^a^	**5.54 ± 0.95**	**5.24 ± 0.61**^b^
1.Champions exist and strongly support program	5.36 ± 0.81	6.00 ± 1.41	5.43 ± 1.22	5.40 ± 0.97
2.Strong champions with ability to garner resource	5.09 ± 0.83	5.82 ± 1.38	5.43 ± 0.85	5.20 ± 0.79
3.Leadership support from within larger organization	5.27 ± 0.91	5.78 ± 0.94	5.71 ± 0.99	5.50 ± 0.97
4.Leadership support from outside of organization	5.09 ± 0.83	5.41 ± 0.80	5.50 ± 1.00	5.00 ± 0.67
5.Strong public support	4.73 ± 1.27	5.28 ± 0.96	5.14 ± 1.03	5.10 ± 0.88
**b) Funding Stability**	**4.82 ± 0.96**	**5.67 ± 0.87**^a^	**5.46 ± 0.93**	**5.07 ± 0.93**
1.Exists in a supportive state economic climate	5.18 ± 0.87	6.06 ± 0.64^a^	6.00 ± 0.78	5.30 ± 0.68
2.Policies to ensure sustained funding	5.00 ± 1.10	5.69 ± 0.79^a^	6.00 ± 1.11^a^	5.00 ± 0.67^bc^
3.Funded through a variety of sources	4.45 ± 1.37	5.25 ± 1.65	4.13 ± 1.69^a^	4.90 ± 1.52^bc^
4.A combination of stable and flexible funding	4.55 ± 1.29	5.50 ± 1.10	5.67 ± 1.11	5.00 ± 1.16
5.Sustained funding	4.91 ± 0.94	5.78 ± 1.06	5.67 ± 1.40^a^	5.33 ± 1.23
**c) Partnerships**	**4.92 ± 0.77**	**5.31 ± 0.62**^a^	**5.39 ± 0.82**	**5.16 ± 0.69**
1.Diverse organizations are invested in program	5.27 ± 0.65	5.39 ± 0.61	5.69 ± 0.86	5.50 ± 0.85
2.Community leaders involved with the program	4.64 ± 1.12	5.00 ± 0.89	4.85 ± 1.21	4.90 ± 0.74
3.Community members committed to program	5.20 ± 1.03	5.56 ± 0.78	5.53 ± 0.83	5.20 ± 0.92
4.Program communicates with community leaders	4.73 ± 0.91	5.00 ± 1.00	5.15 ± 0.69	5.10 ± 0.88
5.Community engaged in the goal development	4.90 ± 0.99	5.33 ± 0.69	5.40 ± 1.18	5.10 ± 0.74
**d) Organizational Capacity**	**5.42 ± 0.79**	**5.91 ± 0.63**	**5.80 ± 0.89**	**5.38 ± 0.68**
1.Program well integrated into the NGO operations	5.27 ± 1.01	5.78 ± 0.88	5.73 ± 1.10	5.50 ± 0.71
2.Organization systems support program needs	5.55 ± 0.93	6.06 ± 0.73	5.93 ± 0.88	5.40 ± 0.70
3.Leadership articulates program vision to external	5.45 ± 0.82	5.82 ± 0.64	5.80 ± 0.86	5.30 ± 0.82^b^
4.Leadership efficiently manages staff resources	5.45 ± 0.82	5.94 ± 0.83	5.87 ± 0.92	5.40 ± 0.84
5.Adequate staff to complete goals	5.36 ± 0.92	5.94 ± 0.73	5.67 ± 1.23	5.30 ± 0.95
**e) Program Evaluation**	**5.38 ± 0.74**	**5.81 ± 0.48**	**5.89 ± 0.69**	**5.52 ± 0.69**
1.Capacity for quality program evaluation	5.45 ± 0.69	5.89 ± 0.68	5.80 ± 0.78	5.40 ± 0.84
2.Reports short term and intermediate outcomes	5.27 ± 0.79	5.89 ± 0.68	5.87 ± 0.83	5.50 ± 0.71
3.Results inform program plan and implement	5.50 ± 0.85	5.72 ± 0.58^a^	5.80 ± 0.86	5.80 ± 0.63
4.Results to tell successes to funders and others	5.50 ± 0.97	5.78 ± 0.73	6.07 ± 0.80	5.50 ± 0.85
5.Provides evidence to public that program works	5.38 ± 1.06	5.78 ± 0.55	5.93 ± 0.88	5.40 ± 1.08
**f) Program Adaptation**	**5.02 ± 0.75**	**5.75 ± 0.58**^a^	**5.64 ± 0.92**	**5.12 ± 0.74**^b^
1.Periodically reviews the evidence base	5.00 ± 1.34	6.00 ± 0.69^a^	6.07 ± 0.96	5.60 ± 0.84^b^
2.Adapts strategies as needed	5.45 ± 0.82	5.67 ± 0.69^a^	5.87 ± 0.83^a^	5.30 ± 0.68
3.Adapts to new science	4.55 ± 1.21	5.78 ± 0.65	5.71 ± 0.83	5.00 ± 0.71
4.Proactively adapts to changes in the environment	5.09 ± 0.70	5.67 ± 0.69^a^	5.73 ± 1.10^a^	5.00 ± 1.23^bc^
5.Makes decisions about ineffective components	4.89 ± 0.93	5.59 ± 0.94^a^	5.07 ± 1.86	4.78 ± 0.97
**g) Communications**	**5.27 ± 0.84**	**5.61 ± 0.57**	**5.68 ± 0.58**	**5.06 ± 0.70**^b^
1.Communication strategies secure public support	5.09 ± 0.83	5.44 ± 0.92	5.40 ± 0.74	4.90 ± 0.99^bc^
2.Program staff notice program need to public	5.18 ± 0.98	5.61 ± 0.70	5.79 ± 0.70	5.00 ± 0.67
3.Marketed in a way that generates interest	5.27 ± 0.91	5.72 ± 0.67	5.67 ± 0.62	5.00 ± 0.94^bc^
4.Increases community awareness	5.45 ± 1.04	5.67 ± 0.69	5.80 ± 0.86	5.20 ± 0.79^b^
5.Demonstrates value to the public	5.36 ± 0.92	5.61 ± 0.61	5.80 ± 0.68	5.20 ± 0.63
**h) Strategic Planning**	**5.36 ± 0.64**	**5.56 ± 0.79**	**5.51 ± 0.87**	**5.10 ± 0.60**^c^
1.Future resource needs	5.36 ± 0.67	5.72 ± 0.75	5.79 ± 0.80	5.20 ± 0.63
2.Long-term financial plan	5.18 ± 0.75	5.25 ± 1.34	5.07 ± 1.22	4.60 ± 1.17
3.Sustainability plan	5.36 ± 0.92	5.44 ± 0.98	5.36 ± 0.84	4.80 ± 0.79
4.Goals understood by all	5.55 ± 0.82	5.61 ± 0.78	5.53 ± 0.92	5.50 ± 0.71
5.Clearly outlines roles and responsibilities	5.36 ± 1.03	5.67 ± 0.77	5.60 ± 0.99	5.40 ± 0.70

One-Way ANOVA and pairwise multiple comparisons with Bonferroni correction were used:

^a^Significant difference in the mean score when compared to that in Round 1

^b^Significant difference in the mean score when compared to that in Round 2

^c^Significant difference in the mean score when compared to that in Round 3

**Fig. 1:**
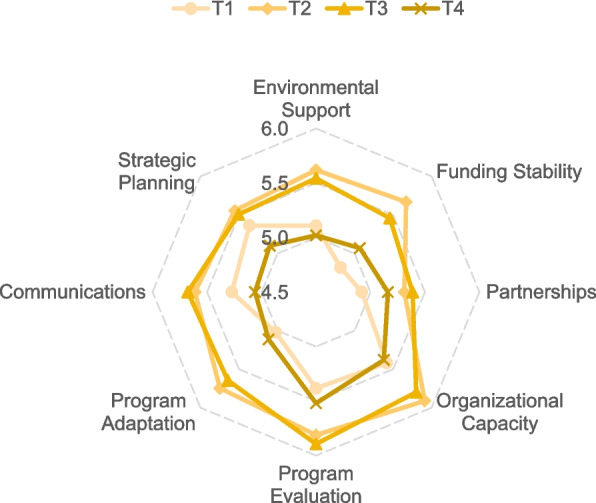
Sustainability capacity score by domains across 4 project years T1: Year 2018; T2: Year 2019; T3: Year 2020; T4: Year 2021

The dynamics of the four-round performance of each PSAT domain are presented in Fig. 1. Domains that were consistent facilitators across the three-year implementation were organisational capacity (5.91 ± 0.63, 5.80 ± 0.89, and 5.38 ± 0.68, respectively) and programme evaluation (5.81 ± 0.48, 5.89 ± 0.69, and 5.52 ± 0.69, respectively). In contrast, consistent barriers were the domains partnerships (5.31 ± 0.62, 5.39 ± 0.82, and 5.16 ± 0.69, respectively), and strategic planning (5.56 ± 0.79, 5.51 ± 0.87, and 5.10 ± 0.60, respectively). Environmental support was a barrier (5.65 ± 0.92 and 5.54 ± 0.95, respectively) at the first two programme years but a facilitator (5.24 ± 0.61) in the final year. Funding stability was a facilitator in the first year (5.67 ± 0.87) but became a barrier in the last two years (5.46 ± 0.93 and 5.07 ± 0.93, respectively). Programme adaptation was a facilitator in the first two years (5.75 ± 0.58 and 5.64 ± 0.92, respectively) and a barrier in the final year (5.12 ± 0.74), and communications were found as a barrier in the first and final year (5.61 ± 0.57 and 5.06 ± 0.70, respectively) but a facilitator in the second year (5.68 ± 0.58). The following section and Table 3 illustrate each PSAT domain based on both qualitative and quantitative results.

**Table 3:** Barrier and facilitator themes by PSAT domains

No	PSAT domains	Facilitator theme	Barrier theme
1	Environmental Support	Leadership endorsement and encourage Implementation leads’ support	Lack of endorsement from the government Local attitudes towards intergeneration relationship Implementation leads’ ability to manage the resources
2	Funding Stability	Support from commissioner	Fixed term of the funding Lack of funding sources after the seed donation Lack of funding source to adapt to changing environment
3	Partnership	Effective use of existing recruitment channel and member pool Partner with academics	Lack of coordination with schools and other NGOs Limited network to recruit youth Competition between similar program for recruitment of participants Lack of involvement of community members for co-creation
4	Organizational Capacity	Professionalism and motivation of colleagues Mission alignment Physical structure fits program implementation High autonomy for program implementation Learning culture within the organization High priority of the program Adequate staffing level	Training for new staff is lacking Lack of clarification of the program vision to external partners Time constraints during pandemic
5	Program Evaluation	Support from the university for conducting evaluation Annual report for program outcomes though multiple channels	Survey design is inappropriate for older adults Time constrains to collect data during pandemic
6	Program Adaptation	Adapt program design to meet schedule and availability of participants	Rigid protocol for program design Difficult to adapt program implementation to critical incidents, such as pandemic and social movement Difficult to adapt program to unexpected participant conditions Lack of knowledge of adapting program design to the relative theories
7	Communications	Outcome observed Communicate with participants to make sure they understand the objective of the program	Unable to effectively communicate with participants due to pandemic Misalignment between NGOs and participants’ perspectives Lack of marketing to promote the program Unattractive program design
8	Strategic Planning	Clearly outlines roles and responsibilities for all stakeholders	Concerns on the sustainability of the program Lack of long term financial plan Lack of plans for resource management

### Environmental support

This domain was rated as barriers in the first two years but a facilitator at the last year. Among five relative items, “the programme has strong public support” (Year 1–3: 5.28 ± 0.96, 5.14 ± 1.03, 5.10 ± 0.88) and “the programme has leadership support from outside of the organization” (Year 1–3: 5.41 ± 0.80, 5.50 ± 1.00, 5.00 ± 0.67) were the ones with consistent barriers. Particularly, staff mentioned that society’s awareness of the importance of intergenerational relationship, and public attitude towards intergenerational relationships can be challenges to implement the programme:

“It is a long process to change local attitudes. People won’t feel intergeneration is an impotent topic suddenly.” (AS9)

Despite it, this project was held under a supportive internal environment, reflected by a consistent facilitator of “the programme has leadership support from within the larger organization” (Year 1–3: 5.78 ± 0.94, 5.71 ± 0.99, 5.50 ± 0.97). For example, staff mentioned that they received support from their mid and high-level leadership, and the programme was a priority in their organizations:

“Three leaders were all very supportive to my work. I think the most important thing is their support and trust. They usually provide insights that I cannot think of” (AS4)

“I can see this project is a priority in my organization. If I need space for hosting activities, the organization would prioritize it to us first.” (BS7)

Additionally, “champions exist who strongly support the programme” was a facilitator except for the second year (Year 1–3: 6.00 ± 1.41, 5.43 ± 1.22, 5.40 ± 0.97). Each organization employed a designated implementation lead and they were perceived as supportive based on staff interviews:

“[The lead] would provide detailed suggestions for activity design and be involved in the front-line each time of the big event.” (AS5)

However, the programme “has strong champions with the ability to garner resources” became a consistent barrier after the first year and the score continuously decreased (Year 1–3: 5.82 ± 1.38, 5.43 ± 0.85, 5.20 ± 0.79). For example, some interviewees mentioned that the implementation lead changed to another person who is brand new to this programme; therefore cannot provide enough support for resource allocation for them.

### Funding stability

Funding stability of JCGCP was perceived at barriers, except for the first year. The item with consistently lowest scores in this domain was “the programme is funded through a variety of sources” (Year 1–3: 5.25 ± 1.65, 4.13 ± 1.69, 4.90 ± 1.52). However, the funding was considered sustained for this programme (Year 1–3: 5.78 ± 1.06, 5.67 ± 1.40, 5.33 ± 1.23), which was a consistent facilitator across the whole programme period. In addition to it, a barrier of “having a combination of stable and flexible funding” existed at the first and third year. Fluctuations also existed in the scoring of “the programme implements policies to help ensure sustained funding” (Year1-3: 5.69 ± 0.79, 6.00 ± 1.11, 5.00 ± 0.67), starting with high scores in the start and drop at the end of the project. This may be because the whole programme was funded through one specific external donor for a fixed term to three NGOs. Based on the interview data, the staff didn’t mention other funding sources and was afraid that the programme would be terminated in their organization if the donor discontinues the funding:

“Unless the donor puts extra effort to fund this programme at a larger scale, otherwise, I don't think it will survive if no funding coming in.” (AS1)

Furthermore, there was no flexible funding to provide an attractive salary and try new activities out of the original proposal:

“Because I want to try something new, which requires more effort or more trials. I have to reply on external donors, but these are difficult.” (CS2)

“The current funding scheme does not support me to increase salary for programme contracted staff while staff affiliated with the government can get the salary increase because of the policy. It’s hard to maintain low staff turnover.” (AS2)

### Partnerships

Partnerships was a consistent barrier across the project period. Items with lowest scores were “community leaders are involved with the programme” (Year 1–3: 5.00 ± 0.89, 4.85 ± 1.21, 4.90 ± 0.74) and “the programme communicates with community leaders” (Year 1–3: 5.00 ± 1.00, 5.15 ± 0.69, 5.10 ± 0.88). Based on interview results, staff mentioned that lacking enough government involvement to sustain the programme:

“Our organization don’t’ have strong networks with local communities and schools, you know. So, when we reached out, the response we received wasn’t very good.” (CS3)

Additionally, “community members are passionately committed to the programme” (Year 1–3: 5.56 ± 0.78, 5.53 ± 0.83, 5.20 ± 0.92) and “the community is engaged in the development of programme goals” (Year 1–3: 5.33 ± 0.69, 5.40 ± 1.18, 5.10 ± 0.74) were also perceived as barriers across the whole programme period. Barriers were listed as a lack of network to reach and maintain potential participants such as schools and other youth organizations. In addition, some participants mentioned that there was a lack of co-creation methods involving older adults and youth when designing the programme:

“The thing is we need to get school leaders and parents’ buy-in instead of youth. However, their current focus is study performance instead of intergeneration relationship.” (CS2)

“I think it’s better to create opportunities for youth and older adults to get involved in programme design to make the programme attractive and fulfil their needs.” (CS8)

On the opposite, strengths existed in investment from diverse organizations (Year1-3: 5.39 ± 0.61, 5.69 ± 0.86, 5.50 ± 0.85). In staff interviews, facilitators were mentioned as having close relationship with academics, funders, and sister institutes within the large organization:

“I think the university provides a lot of support in terms of theory-informed programme design, programme evaluations, and on-site implementation support. The funder is also supportive and responsive to our requirement.” (AS1)

### Organizational capacity

Organizational capacity was regarded as one of the facilitators consistently presented across three years. The item with the highest scores was “Organization systems are in place to support programme needs” (Year1-3: 6.06 ± 0.73, 5.93 ± 0.88, 5.40 ± 0.70). Additionally, well integration of the programme into daily operations, leadership’s capability to communicate clearly and manage resources, and adequate staffing were all considered facilitators across the programme life-cycle. In staff interviews, facilitators were mentioned as compatibility between the programme and organization structure and mission; learning culture at institutional levels; and staff’s motivation, collaborative efforts, and skills:

“Our organization already has a similar service model in place. For example, our youth service department has a comprehensive service team to provide services to older adults.” (CS8)

“Our organization’s ultimate goal is to bring public attention to similar social issues and the needs of older adults.” (AS4)

“I think great things need to be explored. I will only provide some insights and let the front-line staff to trial and explore.” (BS1)

However, when compared the sustainability capacity of final year to the baseline, all items were dropped except for “programme well integrated into NGO’s operation”. Based on the staff interviews, several barriers were identified as lack of clarification of the programme vision to external partners, lack of continuing training for new staff, and conflict in managing time, administration and logistics (e.g., refund, venue, manpower) of programmes:

“The participant didn’t understand the real objective of the programme. Many youth perceived themselves as volunteers.” (AS7)

“Manpower was not enough during pandemic. I need to hold three rounds of activates in one day, which is really a pressure.” (BS8)

### Programme evaluation

Programme evaluation was another consistent facilitator. The item “the programme reports short-term and intermediate outcomes”, “has the capacity for quality programme evaluation”, “evaluation results inform programme planning and implementation”, and “evaluation results are used to demonstrate successes to funders, key stakeholders, and the public”, were all rated at high scores across the three-year implementation. Staff mentioned in interviews that support from partner university was important for programme evaluation. In addition, multiple channels were applied to inform the public and key stakeholders about the positive impact such as annual capacity-building workshop, programme symposium, public showroom, and online social media.

However, the “the programme has the capacity for quality programme evaluation” in year 3 was lower than the baseline. Staff mentioned that the questionnaire design was inappropriate for those older adults with lower cognitive functions. In addition, limits in manpower (especially under the impact of COVID-19 pandemic and social movement) to collect data was also an important barrier during programme evaluation:

“the questionnaire is too long. Older adults don’t have patience to finish the whole thing. I don’t know whether their responses are accurate or not” (CS5)

### Programme adaptation

Programme adaption started from being a facilitator but transferred to a barrier at the final year. The item with lowest scores across the project years was “the programme makes decisions about which components are ineffective and should not continue” (Year1-3: 5.59 ± 0.94, 5.07 ± 1.86, 4.78 ± 0.97). Staff mentioned in the interview that the programme adaptability is a barrier:

“The programme has high rigidity, we cannot make adaptations because it has to meet specific research requirement.” (CS1)

The item “the programme periodically reviews the evidence base” (Year1-3: 6.00 ± 0.69, 6.07 ± 0.96, 5.60 ± 0.84) and “the programme adapts strategies as needed” (Year 1–3: 5.67 ± 0.69, 5.87 ± 0.83, 5.30 ± 0.68) were consistent facilitators in the whole implementation years. Staff recognized the improvements in project implementation by years, with strategies such as delivery mode adaptations based on environmental changes and marketing strategy adjustments to recruit participants:

“Based on last year’s experience, we advanced our schedule for the promotion work in schools” (BS2)

Other items with mixed impacts were “the programme adapts to new science”, and “the programme proactively adapts to challenges of the environment”, being facilitators at the beginning but inverse to barriers in the end. In staff interviews, the frequently mentioned barriers were difficulties in understanding and applying the theoretical framework in activity design, strikes of COVID-19 pandemic and social movements on implementation, and difficulties in controlling participants’ conditions, such as schedules of youth and older adults’ health conditions:

“I think there was a gap between theories and real practice. I found myself really hard to understand the key points and apply the knowledge learnt from the workshop into the daily work.” (AS3)

“The pandemic and social movement make the implementation really hard. Older adults lost trust in the youth because of the social movement and they are afraid of going out to participate in the activity because of the pandemic.” (AS1)

### Communications

The impact of communications were perceived fluctuant, with below-average scores in the first and last years, and above-average scores in the second year. The item with lowest score across three years was “the programme has communication strategies to secure and maintain public support” (Year1-3: 5.44 ± 0.92, 5.40 ± 0.74, 4.90 ± 0.99). In qualitative interviews, staff mentioned that eMarketing is not enough to catch public’s attention:

“I think the partner university can put more effort to help with promoting this programme to the public via social media such as filming videos.” (AS3)

Four items with significant drops in performance in the last year were “programme staff communicate the need for the programme to the public” (Year1-3: 5.61 ± 0.70, 5.79 ± 0.70, 5.00 ± 0.67), “the programme is marketed in a way that generates interest” (Year1-3: 5.72 ± 0.67, 5.67 ± 0.62, 5.00 ± 0.94), “the programme demonstrates its value to the public” (Year1-3: 5.61 ± 0.61, 5.80 ± 0.68, 5.20 ± 0.63), and “the programme increases community awareness of the issue” (Year 1–3: 5.67 ± 0.69, 5.80 ± 0.86, 5.20 ± 0.79). These were reflected in staff interviews that lack of understanding of participants’ needs, unattractive programme design, and difficulties in communication with participants during pandemic were barriers in implementation:

“I don’t think current programme design caters for youth’s needs and specific personalities.” (CS2)

“Before the pandemic, we usually invite older adults come to our centre to gather their feedback, build good relationship with them, but now we cannot do it.” (BS6)

### Strategic planning

Strategic planning was another consistent barrier across the programme life cycle. Specifically, the item with lowest rating across years was “the programme has long-term financial plan” (Year 1–3: 5.25 ± 1.34, 5.07 ± 1.22, 4.60 ± 1.17) and “sustainability plan” (Year 1–3: 5.44 ± 0.98, 5.36 ± 0.84, 4.80 ± 0.79). Items with mixed impact were “programme’s goals are understood by all stakeholders” and “plans for future resource needs”. Staff mentioned deficiencies in planning, such as:

“I think the most important thing is to set up a very clear goal and make sure everyone is on the same page.” (AS1)

“I think it is better to have a implementation plan, such as the specific schedule for each task, the potential difficulties and action plans.” (AS3)

“I think a prior plan for resource allocation is important, such as when and where should I book the space.” (BS1)

However, “the programme clearly outlines roles and responsibilities for all stakeholders” has a positive impact across the three programme years (Year 1–3: 5.67 ± 0.77, 5.60 ± 0.99, 5.40 ± 0.70). Based on the staff interview, each interviewee can clarify their roles in this programme including the changes of their roles.

## Discussion and implications

This study evaluated the sustainability capacity, along with the associated facilitators and barriers, of a three-year community-based intergenerational project in Hong Kong. The evaluation over the baseline year and 3 years of implementation reflected the life-cycle of the project: the programme sustainability capacity started at a relatively low level, increased as implementation progressed, and subsequently declined towards the end. By domains, we found that organisational capacity and programme evaluation were consistent facilitators, while partnerships and strategic planning emerged as barriers. Additionally, funding stability, environmental support, programme adaptations, and communications exhibited mixed effects during the course of implementation.

The findings of our study corroborate established patterns in the program sustainability literature while also introducing context-specific insights. Consistent with prior studies on sustaining community health initiatives, we found that strong organizational capacity and ongoing program evaluation served as key facilitators of sustainability [31]. This aligns with the broader consensus that robust infrastructure, staff support, and evidence of impact bolster a program’s ability to endure [32]. Conversely, persistent challenges with funding stability, forging partnerships, and strategic planning in our project echo well-documented barriers in similar programs [33]. Indeed, deficits in public support or external environmental support were evident in our case, mirroring observations that novel community initiatives often struggle to gain early societal buy-in [34]. The mixed performance of domains like communications and adaptation over time further reflects patterns noted in existing literature that sustainability capacity is not static, but fluctuates over a program’s life cycle in response to evolving internal and external conditions [35].

Our results showed that organizational capacity and program evaluation emerged as consistent facilitators of sustainability throughout the project. This suggests that the program’s strong internal infrastructure for implementation and its rigorous evaluation processes played a critical role in sustaining the intervention. The organizational capacity might be strengthened by holding capacity development workshops annually. The program provides opportunities to gather a wide range of partners and stakeholders and different parties could co-design, co-engage, and co-supervise activities, as well as co-create and share knowledge and evidence on intergenerational wellbeing. After the workshops, all participators also performed a higher commission in the organisation, implementation and evaluation of programmes, which was indispensable for the success of the project. Regarding program evaluation as a consistent facilitator, this may be attributed to the program’s commissioning of an academic institution to carry out pre-test and post-test design to evaluate the effects of intergenerational interventions. Evaluation of the implementation and sustainability capacity of the project was also conducted annually. The results of the evaluation were disseminated periodically through workshops and symposiums to all stakeholders and the public. These measures guaranteed timely evaluation, monitoring and information delivery of the process and outcomes of the JCGCP programme.

This study also identified deficiencies in sustainability capacity when implementing a community-based intergenerational programme. First, regular evaluation and monitoring were necessary across the whole course of the programme, and challenges and emphasis can be different by implementation phases. In the initial phase, focus can be put on the funding stability and partnerships. Barriers to funding were well identified and recognised in other projects, especially those with short-terms [36]. Communication, trust and collaboration between partners, participants and within organisations were also vital for success in implementation and sustainability [37–39]. In the middle phase, partnership, communication, adaption, and strategic planning may require more attention. Major challenges include: 1) strategies to attract, recruit and maintain participants; 2) adaptions to external environment and health conditions and personal requirements of participants; 3) consensus among different stakeholders, including partners, participants, and commissioners; and 4) continuous training and support in the face of personnel change. In the late phase of programme, focus can be put on funding stability, environment support, communication and strategic planning of the programme. In this phase, internal and external support decreased by the end of existing project. Preparation and long-term strategic plans for the future programme were needed [40], including: 1) dissemination of evidence of benefits and value of the programmes for all potential audience in community; 2) maintenance and enhancement of interest, communication, and participation of different stakeholders; 3) exploration of potential resource, community support and external funding for the spread and routinisation of activities; and 4) being an integral part of the social and health service system in community.

Our findings highlighted several key issues related to intergeneration dynamics that are important for assessing the sustainability capacity of intergenerational programme. These include: 1) knowledge on the concept and theoretical framework of intergeneration, and perceived value of intergenerational activities and harmony, especially for frontier staff and programme participants; 2) good communication, interaction and synergy between younger and older participants; 3) adequate arrangement and synergy of services for both youth and elderly; and 4) identification of reciprocal roles (rather than unilateral contribution) by younger and older participants in intergenerational activities. For future evaluations of sustainability capacity of intergenerational programmes, it is essential to involve a broader range of stakeholders, such as funders, commissioners, project partners, community, and programme recipients. Furthermore, continuous and long-term assessments across various categories of intergenerational programmes, utilizing PSAT items and domains, are necessary to gain a comprehensive understanding of strengths and shortcomings, guding improvements for specific interventions [38].

The fluctuating sustainability capacity during the course of programme implementation reflected that the single strategy, the capacity-building workshop, may not be sufficient. To enhance the sustainability capacity, partnerships and strategies planning could be the starting point. Based on a preceding literature mapping the PSAT domains to The Expert Recommendations for Implementing Change (ERIC) taxonomy [41], six types of strategies could be adopted in our community context to address the consistent barriers across the programme life cycle: 1) provide more enhanced training to stakeholders to plan for sustainability; 2) develop stakeholder interrelationships by setting up programme committee, holding consensus meetings, and building coalitions among diverse stakeholders; 3) provide interactive ongoing technical assistance for the course of implementation; 4) adapt and tailor to context by performing continuous monitoring; 5) use financial strategies, and 6) use evaluative and iterative strategies to assess readiness and local needs, and communicate positive outcomes. Those strategies could be further investigated in future study.

### Strengths and limitations

This was one of the few studies that assessed the sustainability capacity of a community-based intergenerational project. The study has several strengths. First, we used a reliable tool, PSAT, to assess the programme sustainability. This tool as been widely used for a variety of community and public health programmes [42]. Secondly, we used a combination of quantitative and qualitative methods, which was beneficial for the reliability and extension of the findings [43]. We also employed a multi-time-points design and conducted the evaluation in each implementation year. This approach revealed the changes in the sustainability capacity and associated facilitators and barriers across the life cycle of the intergenerational programme. It also helped monitor and timely improve the implementation of the programme. Third, this study focused on the community-based intergenerational programme, providing some new insights into the sustainability capacity specific to this type of project.

This study also has several limitations. First, the sample size was limited, which inherently limits the statistical power of our analysis and the generalizability of the results. We did not conduct a formal power analysis as the participant pool was fixed by the program structure and our priority was to include all available providers and gain in-depth insights into the program’s sustainability rather than to achieve a large, statistically powered sample. Consequently, the quantitative findings are offered as preliminary and descriptive. We acknowledge that the limited sample size does not meet typical methodological expectations and that the results should be interpreted with caution. In addition, the participants were different in each year of the assessment due to staff turnover. This hindered longitudinal observations of a consistent cohort, limiting comparisons across different assessment points. Thus, it is not possible to definitely determine whether the changes in PSAT scores over the years were due to actual differences or respondent bias. Second, this study was conducted from the perspectives of service providers, lacking views from other stakeholders (funders, commissioners, assessors, programme participants) on the sustainability of programme. Finally, this study was limited in analyses of the sustainability capacity of different types of activities because the study design focused on assessing the overall sustainability of the project. The JCGCP consisted of three types of intergenerational activities: life review, medical visit buddy, and traditional culture and health programmes. The participants, content, organisation, venue and activity settings also varied across them. Therefore, future research should administer assessments at the programme-specific level to allow direct comparisons across different intervention strategies and provide more targeted recommendations for improvement.

## Conclusion

JCGCP was a three-year community-based intergenerational project to promote intergenerational harmony, solidarity and welling. The perceived changes of sustainability capacity over the three-year implementation reflected the life cycle of programme. Facilitators for sustainability were organisational capacity and programme evaluation. Barriers were funding stability, environmental support, partnerships, and strategic planning, which were important factors needed to be addressed in the long-term planning and implementation.

## Supplementary Information

Additional file 1. Journal Article Reporting Standards for Mixed Methods Research.

Additional file 2. Descriptions of eight domains in PSAT.

## Data Availability

The datasets used and/or analysed during the current study are available from the corresponding author on reasonable request.

## Abbreviations

PSAT: Program Sustainability Assessment Tool
JCGCP: Jockey Club Generation Connect Project
NGOs: Non-governmental organisations
LR: Life Review
MVB: Medical Visit Buddy
TC: Traditional Culture and Health Programmes
