# Supplementary material for: Evaluating the sustainability of a three-year community-based intergenerational project in Hong Kong: a longitudinal mixed methods study from the perspective of service providers
Source: Implement Sci Commun. 2026 Apr 15;7:105. doi: 10.1186/s43058-026-00920-3 (PMC13220636; doi:10.1186/s43058-026-00920-3)
Supplement: Supplementary file 2 — Additional file 2. Descriptions of eight domains in PSAT. [file 43058_2026_920_MOESM2_ESM.docx]

**Appendix 2. Descriptions of eight domains in PSAT**

| **Sustainability domain** | **Description** |
| --- | --- |
| Environmental Support | Having a supportive internal and external climate for your program |
| Funding Stability | Establishing a consistent financial base for your program |
| Partnerships | Cultivating connections between your program and its stakeholders |
| Organizational Capacity | Having the internal support and resources needed to effectively manage your program |
| Program Evaluation | Assessing your program to inform planning and document results |
| Program Adaptation | Taking actions that adapt your program to ensure its ongoing |
| Communications | Strategic communication with stakeholders and the public about your program |
| Strategic Planning | Using processes that guide your program’s direction, goals, and strategies |
